# Sleep-Disordered Breathing in Newly Diagnosed Patients of Lung Cancer

**DOI:** 10.7759/cureus.25230

**Published:** 2022-05-23

**Authors:** Shrikant Bhaisare, Rajnish Gupta, Jitendra Saini, Amartya Chakraborti, Sagar Khot

**Affiliations:** 1 Department of Pulmonary Medicine, National Institute of Tuberculosis and Respiratory Diseases, New Delhi, IND

**Keywords:** obstructive sleep apnea, non-invasive ventilation, lung cancer, polysomnography, sleep-disordered breathing

## Abstract

Sleep-disordered breathing (SDB) is highly prevalent in patients with cancer and affects their prognosis. However, data on SDB in lung cancer patients are lacking, and few studies have conducted level I polysomnography (PSG) in this patient population. This study aimed to measure SDB in newly diagnosed lung cancer patients at the sleep clinic of a tertiary respiratory institute in New Delhi, India, for eight months. This study included 30 patients. Participants received a clinical examination, completed a sleep questionnaire, and then underwent overnight PSG. We scored sleep parameters according to the American Academy of Sleep Medicine guidelines. Both descriptive and inferential statistics were used to analyze the data. We used univariate analysis with chi-square testing, and p<0.05 was considered significant. SDB and obstructive sleep apnea (OSA) were found in 66.6% and 56.6% of patients, respectively. Mild, moderate, and severe OSA were seen in 26.6%, 16.6%, and 13.3% of patients, respectively. Nocturnal oxygen desaturation (NOD) or NOD90 (i.e., when >30% of sleep time was spent with oxygen saturation levels <90%) was seen in 13.3% of patients. Adenocarcinoma was the most common histological variant of cancer. Tumor-node-metastasis staging was significantly associated with the presence of OSA (p=0.045). Lung cancer patients should receive routine PSG to identify and manage patients with SDB, especially given that symptoms of SDB such as easy fatigability and non-refreshing sleep are overlooked as symptoms of lung cancer. Proper management of SDB or OSA would help improve patients' quality of life and improve their overall prognosis.

## Introduction

Lately, the association between sleep-disordered breathing (SDB) and cancer has been of interest. Studies show a high prevalence of SDB in cancer patients, occurring in up to 49% of cases [1-2]. Marshall et al. reported that in a 20-year study, patients with an apnea-hypopnea index (AHI) of ≥15 had 3.4 times the risk of developing lung cancer than patients without obstructive sleep apnea (OSA) [3]. SDB is also associated with increased mortality rates in cancer patients. Nocturnal oxygen desaturation (NOD) or NOD90 (i.e., when >30% of sleep time is spent with oxygen saturation levels <90%) is strongly associated with cancer mortality, highlighting the importance of hypoxemia in patients with lung cancer [4]. Intermittent hypoxia is a trigger for tumor growth and progression [5]. Cells intermittently exposed to hypoxia are more resistant to radiation and apoptosis and are more prone to metastasis [6]. However, the association between SDB and poor outcomes in all types of cancers has not been well explored. Based on a large nationally representative health insurance database evaluation, OSA increased the risk of pancreatic and kidney cancer and melanoma, but not colorectal, breast, and prostate cancers [7]. There is a paucity of data on the relationship between SDB and lung cancer, with few studies in which polysomnography (PSG) was conducted in a sleep lab with a technician/doctor. Therefore, we conducted this prospective observational study to investigate the existence and pattern of SDB in patients newly diagnosed with lung cancer.

## Materials and methods

We conducted this prospective observational study in the sleep lab of a tertiary hospital in New Delhi for eight months. The study was approved by the Institutional Review Board (NITRD/PGEC/2017/6106), namely the Post Graduate Ethics Committee of the National Institute of Tuberculosis and Respiratory Diseases. All consecutively diagnosed lung cancer patients enrolled in the lung cancer clinic were included in this study if they met the inclusion criteria. The study included patients older than 18 years who were diagnosed with lung cancer irrespective of sleep symptoms and able to perform their day-to-day activities.

Of the 42 patients eligible, 12 were excluded due to poor performance status. Thirty patients were enrolled in the study and provided written informed consent. We recorded detailed clinical histories from each patient with particular regard to sleep concerns, Epworth sleepiness scale (ESS), smoking history, and occupational and environmental exposure to carcinogens. We conducted physical examinations and measured each participant's body mass index (BMI) and neck circumference. We recorded the participant's complete hemogram, kidney function tests, liver function tests, lipid profile, thyroid profile, and electrocardiogram (ECG).

All patients underwent overnight PSG on CompuMedics E-Series PSG machines (CompuMedics Ltd., Abbotsford, Victoria, Australia). A female attendant monitored female participants. The participants underwent an electroencephalogram, electrooculogram, ECG, and chin and leg electromyogram. We recorded nasal airflow (with help of an oronasal thermistor and nasal pressure transducer), thoracic and abdominal wall movements, transcutaneous oxygen saturation, snoring, and body position. We used manual scoring for all measurements. We assessed apnea, hypopnea, respiratory effort related arousals, respiratory disturbance index, AHI, sleep hypoventilation, NOD, arousal index, and periodic limb movement of sleep (PLMS) index. OSA was defined as AHI > 5. All grading was done according to the American Academy of Sleep Medicine (AASM) guidelines version 2.4 [8]. The lung cancer clinic in our institute addressed lung cancer staging and management plans. The staging was done according to the National Comprehensive Cancer Network lung cancer guidelines version 4 2016 [9].

Statistical analysis

The measurements were collected on a predesigned proforma and gathered into spreadsheet software. We analyzed the data using R version 3.3.2 [10]. Continuous variables were represented as the median plus interquartile range (IQR) for non-normal distribution and as mean plus standard deviation (SD) for normal distribution. Categorical variables were expressed using percentages. We used chi-square tests, Fisher's exact test, and t-test for means, and Mann-Whitney-U tests where applicable. We considered p<0.05 as statistically significant.

## Results

Table 1 presents patient demographic information.

**Table 1:** Patient demographic and health data COPD, chronic obstructive pulmonary disease

Patient characteristics	n (%)
Sex	
Male	26 (87%)
Female	4 (13%)
Histology	
Adenocarcinoma	17 (57%)
Squamous cell carcinoma	12 (40%)
Small cell carcinoma	1 (3%)
Tumor staging	
I	0
II	0
III	19 (66.6%)
IV	11 (33.3%)
Addiction history	
Smokers	26 (86.6%)
Alcohol	9 (30%)
Comorbidities	
Diabetes	2 (6.6%)
COPD	1 (3.3%)
Ischemic heart disease	5 (16.6%)
Hypertension	4 (13.3%)

The study included 26 (87%) men and four (13%) women, and the median age of the study population was 55 years (IQR: 12 years). Patients had a mean BMI of 19.4 ± 3.9 kg/m^2^. Twenty-five patients (84%) had a BMI of <22.9 kg/m^2^, while 14 (46.7%) had a BMI of <18.5 kg/m^2^. Fifteen patients (50%) had a history of occupational exposure to organic dust and smoke, and 10 patients (33.3%) lived in industrial areas, making them prone to exposure to environmental carcinogens and pollution. The most common form of lung cancer was adenocarcinoma (n=17, 56.7%), followed by squamous cell carcinoma (n=12, 40%) and small cell lung cancer (n=1, 3.3%). Sleep and respiratory parameters for the study population are presented in Table 2.

**Table 2:** Distribution of sleep parameters on polysomnography REM, rapid eye movement; NOD, nocturnal oxygen desaturation; ODI, oxygen desaturation index; AHI, apnea-hypopnea index; PLM, periodic limb movement; SD, standard deviation

Parameter	Mean + SD	Range
Sleep latency (min)	18.23+25.24	0-114.4
Sleep efficiency (%)	80.09+12.05	47.4-97.6
Total sleep time (min)	317.88+69.065	161-457
Staging		
Wake (min)	66.6+41.78	7.5-169
REM (min)	25.97+20.02	0-19.9
Stage N1 (min)	72.3 + 49.63	8.5-195
Stage N2 (min)	137.5+69.35	19-315.5
Stage N3 (min)	82.45+58.49	1.5-212.5
NOD (%)	12.34+ 21.1	0-81.05
ODI/hour	8.39+ 11.45	0-41
Lowest SaO_2_ (%)	84.53+ 8.8	57-95
AHI or RDI/hour	12.01+15.52	0.2-60.1
PLM index/hour	1.09+5.02	0-27.5
Total arousal index/hour	6.95+ 4.00	0.7-15.7
No. of sound events/hour (sleep)	45.19+ 102.14	0-503.2

Of the 30 patients enrolled in our study, 17 (56.7%) patients had OSA, none had central apnea, and four (13.3%) had NOD. Of these four patients, one patient had severe OSA. Therefore, our study's total number of SDB cases was 20 (66.7%). Patients had a mean SDB time with oxygen saturation < 90% of 50.6 minutes (± 76 minutes SD). Patients diagnosed with SDB had a mean ESS of 6.25 ± 2.03. Easy fatiguability (n=27, 90%) was the most common symptom, followed by snoring (n=23, 77%). In the subgroup of patients with OSA, easy fatiguability, snoring, unrefreshing sleep, and excessive daytime sleepiness were seen in 94%, 83%, 75%, and 70% of patients, respectively.

Of the 17 patients diagnosed with OSA, eight (47%) had mild OSA, five (29.4%) had moderate OSA, and four (23.53%) had severe OSA. Table 3 presents the relationship between OSA severity and various histological types of lung carcinoma.

**Table 3:** Lung cancer and obstructive sleep apnea severity

Type of cancer (n=30)	Obstructive sleep apnea				
	Absent (n=13)	Total	Present (n=17)		
			Mild	Moderate	Severe
Adenocarcinoma (n=17)	5 (38.4%)	12 (70.5%)	5 (29.4%)	3 (17.6%)	4 (23.5%)
Squamous cell carcinoma (n=12)	7 (53.8%)	5 (29.4%)	3 (17.6%)	2 (11.7%)	0
Small cell carcinoma (n=1)	1 (7.7%)	0	0	0	0

Twelve (70.6%) patients with OSA were diagnosed with adenocarcinoma, whereas the remaining five (29.4%) were diagnosed with squamous cell carcinoma. Severe OSA was seen in four of the 12 (33.3%) patients with adenocarcinoma.

**Figure 1:**
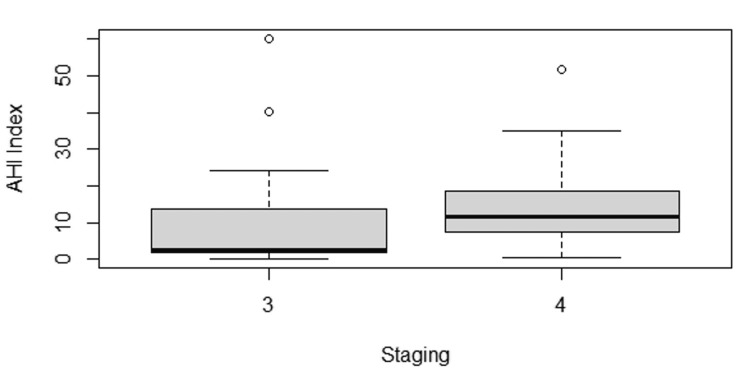
Boxplot of AHI/hour between stage 3 and stage 4 lung cancer patients AHI, apnea-hypopnea index

Lung cancer staging had a statistically significant association with the prevalence of OSA (p=0.045), although its association with the severity of OSA was not significant (p=0.065; Figure 1).

No significant association was found between the histological diagnosis of lung cancer and the severity of OSA (p=0.33). We found no statistically significant association between NOD90 and cancer histologic type or lung cancer staging (p=0.7 and p=0.5, respectively).

## Discussion

We found a high prevalence of SDB among newly diagnosed lung cancer patients, which supports Dreher et al.'s multicenter study that found SDB in nearly half of newly diagnosed lung cancer patients (49%) [1]. Cabezas et al. found that 80% of lung cancer patients had SDB in their at-home sleep apnea study [2]. We found NOD in 13.3% of our patients, an incidence similar to other studies (Abstract: Seijo LM, Perez-Warnisher MT, Cabezas E, Troncoso MF, Gomez T, Melchor R, Pinillos J, El Hachem A, Gotera C, Peces-Barba G, Gonzalez-Mangado N. C110: Prevalence of Obstructive Sleep Apnea Among Volunteers Enrolled in a Lung Cancer Screening Program. Results of the Prospective Sails (Sleep Apnea in Lung Cancer Screening) Study. American Thoracic Society International Conference; May 23, 2017) [1].

OSA is traditionally seen in obese patients with elevated BMI. However, our patient population had a low mean BMI and the cachexia associated with advanced disease process. Despite this, most patients (57%) had OSA. OSA might have an underlying relationship to lung cancer beyond the traditional risk factors for OSA.

Kendzerska et al. reviewed the hospital records of more than 33,000 patients, and, after correcting for factors such as age, gender, addiction, and comorbidities, they reported that patients with severe OSA had a 15% higher risk of developing lung cancer than patients without OSA [11]. NOD was associated with twice the risk of finding positive findings in CT scans during screening compared to those with healthy oxygen saturation levels [12]. NOD90 > 12% was associated with a hazard ratio of 2.33 for developing lung cancer [13]. For our study, we considered NOD90 > 30% as significant. Using NOD90 > 12% as the threshold would have increased the percentage of patients with SDB in our study, further attesting to the association between SDB and lung cancer prevalence. Raising the NOD90 threshold to >30% would help identify the patients more prone to intermittent hypoxemia.

The hypoxic environment in patients with SDB or OSA is a well-known risk factor for lung cancer. Li et al. reported significant alterations in the expression of molecules such as hexokinase 2 (HK2) and glycogen branching enzyme 1 (GBE1) in hypoxic lung cancer cells [14]. HK2 and GBE1 are regulated by hypoxia-inducible factor (HIF), enabling lung cancer cells to invade tissues using focal adhesion kinase activity. Li et al. found a strong correlation between HK2, GBE1, and HIF expression in patients with adenocarcinoma and squamous cell carcinoma [14]. Their expression also correlated with increased tumor-node-metastasis staging in lung cancer, suggesting that the hypoxic stress associated with OSA is related to lung cancer progression and its clinical staging. Huang et al. reported a strong association between AHI and HIF expression (p=0.04) [15]. Li et al. also found a statistically significant association between the severity of OSA, tumor size, and staging (p=0.007 and p<0.001, respectively) [14]. Li et al. also reported a significant difference in survival rates across all lung cancer stages in patients with mild, moderate, and severe OSA (p=0.0061) [14].

The role of positive airway pressure (PAP) to address OSA and improve patient quality of life is well established, but it can also affect disease prognosis. Almost 94% and 75% of OSA patients in our study had easy fatigability and unrefreshing sleep, respectively, symptoms common among advanced stage lung cancer patients that health care professionals might overlook. Advising PAP therapy in such patients could improve those symptoms and alleviate hypoxic stress on the body.

Our study had some important limitations. In addition to using a small sample size, we did not use age- and gender-matched cancer patients without SDB as a control group. However, we compared our data to the scant evidence available in the literature. We did not use spirometry to diagnose chronic obstructive pulmonary disease, which could be a confounding factor in the study. Our study was also limited by using stage III or stage IV lung cancer patients, which may have offset the prevalence of SDB or OSA.

## Conclusions

We conducted this study to identify the prevalence of SDB among recently diagnosed lung cancer patients. SDB was present in more than half of the study participants. Patients with lung cancer should be screened for SDB and offer PAP therapy for appropriate patients to treat OSA. PAP might offer them symptomatic relief, decrease the effects of comorbidities, and reduce further morbidity and mortality.
